# Management of Malaria in Children Younger Than 5 Years Old During Coronavirus Disease 2019 Pandemic in Sierra Leone: A Lesson Learned?

**DOI:** 10.3389/fped.2020.587638

**Published:** 2021-02-10

**Authors:** Danilo Buonsenso, Francesco Iodice, Bianca Cinicola, Francesca Raffaelli, Solia Sowa, Walter Ricciardi

**Affiliations:** ^1^Department of Woman and Child Health and Public Health, Fondazione Policlinico Universitario A. Gemelli IRCCS, Rome, Italy; ^2^Dipartimento di Scienze Biotecnologiche di Base, Cliniche Intensivologiche e Perioperatorie, Università Cattolica del Sacro Cuore, Rome, Italy; ^3^Center for Global Health Research and Studies, Università Cattolica del Sacro Cuore, Rome, Italy; ^4^Institute of Neurology, Fondazione Policlinico Universitario A. Gemelli IRCCS, Rome, Italy; ^5^Department of Pediatrics, Sapienza University of Rome, Policliclinico Umberto I, Rome, Italy; ^6^Dipartimento Scienze di Laboratorio e Infettivologiche, Fondazione Policlinico Universitario Agostino Gemelli IRCCS, Rome, Italy; ^7^Konta Wallah Community Health Center, Port Loko, Sierra Leone; ^8^Sezione di Igiene, Istituto di Sanità Pubblica, Università Cattolica del Sacro Cuore, Rome, Italy

**Keywords:** malaria, COVID-19, SARS-CoV-2, children, Africa

## Abstract

Growing evidences are showing the potential indirect effects of the Coronavirus Disease 2019 (COVID-19) on the health systems of low-resource settings, where diseases such as Tuberculosis, Human Immunodeficiency Virus (HIV) and Malaria represent major killers. Therefore, we performed a retrospective study aimed to evaluate the impact of COVID-19 on Malaria programs in a peripheral region of Sierra Leone, previously involved by the Ebola outbreak in 2015, when malaria care have been impaired since local health systems were overwhelmed by Ebola cases. During COVID-19 in Sierra Leone, we did not notice a significant drop in malaria diagnosis in children, suggesting that a proactive approach in the management of malaria in endemic countries during COVID-19 may have had a positive impact. A comprehensive approach that include also educational activities to sensitize the local population, was useful to guarantee successful malaria diagnosis and treatment, and prevents excess of malaria deaths due to potential disruption of the local health systems related to the SARS-CoV-2 pandemic.

## Introduction

After the first description in China, severe acute respiratory syndrome coronavirus 2 (SARS-CoV-2) spread worldwide and reached Sub-Saharan Africa. In particular, the first cases in Africa have been described around March 2020 (1). In this part of the world, the number of coronavirus disease 2019 (COVID-19) cases and deaths is rising day by day, although not significantly compared with Europe, Asia, and North and South America (2). The reasons for such a difference are still unexplained, but multiple factors can be involved, including race, sex, socioeconomic status, genetic differences (3), and lack of testing points in most areas, particularly in the peripheries.

The arrival and continuous spread of SARS-CoV-2 in Africa are worrying major organizations (including the World Health Organization and major medical journals) because of the direct impact of the viral spread on a weak health system and the indirect effects of lockdown in a weak economic system (4). Preliminary data arrived from Sierra Leone, where we previously highlighted the social consequences of SARS-CoV-2 lockdown, with most people experiencing economic difficulties and losing their jobs (5). However, it was immediately clear that the health system should be paid special attention to the possible effects of the COVID-19 pandemic on other major killers in Africa. During the last decade, African countries greatly improved the care of patients with these conditions, thanks to a coordinated approach including government acts and the support of major international agencies (such as the World Health Organization and non-governmental organizations). The impact on tuberculosis and HIV care has been widely claimed (6), and there is evidence of the negative impact of the pandemic on immunizations (7) and TB services worldwide (8, 9).

Conversely, the impact of COVID-19 on malaria services in low-to-middle income countries (LMICs) is less established. Considering that malaria mainly kills children under 5 years of age, whereas COVID-19 is relatively mild in this group (10), the potential effects of disrupted malaria programs on children's health could be massive. Using spatiotemporal Bayesian geostatistical models, Weiss et al. estimated that, under pessimistic scenarios, COVID-19-related disruption to malaria control in Africa could almost double malaria mortality in 2020 and the following years. However, real-world data from LMICs are still missing (11). Therefore, we performed this retrospective study aimed to evaluate the impact of COVID-19 on malaria programs in a peripheral region of Sierra Leone.

## Methods

To understand the impact of COVID-19 on malaria care, we retrospectively evaluated the gross numbers of children aged 0–60 months diagnosed with confirmed *Plasmodium falciparum* malaria in the Konta Wallah Community Health Center of Port Loko District, Kamasondo Chiefdom, a peripheral government-recognized malaria inpatient and outpatient unit, referral for an area of ~8,000 people. In this setting, malaria is diagnosed either on a clinical basis or confirmed with a rapid antigen test able to detect *P. falciparum* antigen, with no further diagnostic resources available.

We collected the number of patients tested with confirmed *P. falciparum* malaria during the first 5 months of the year 2020 (January, February, March, April, and May) and compared it with the cases reported in 2018 and 2019 during the same months. In Sierra Leone, the first COVID-19 presumptive cases have been documented at the end of March 2020 and lockdown declared in April 2020. In May 2020, the lockdown has been removed, but people were not allowed to move between districts. Because data have been collected during the very first local peak and the lockdown, we did not calculate the sample size and collected all malaria diagnoses made during the three study periods.

The data were collected, as absolute numbers, by the center's health workers in accordance with the usual rules held in Sierra Leone. Cases were retrospectively obtained using the health facility routine activity reporting forms. In many peripheral Sub-Saharan Africa health centers, data are collected by the health facility on a register by crossing off with a pen the type of disease diagnosed, without registering personal data nor more detailed epidemiological data. Peripheral health centers have a registry book with dedicated pages for the registration of diagnoses, where operators include age groups and final diagnosis (7). Eventually, data were transferred on an Excel file.

The study was approved by a local committee composed of those responsible of the malaria unit of the local health center (S.S.), the research team of the Bureh Town Community Hospital of Sierra Leone, the headman of the community, and the older adults of the village, in a similar way to what happens for all the important political and economic decisions in the examined area (n25_may-2020).

Descriptive and statistical analyses have been performed with STATA v16: χ^2^ or Fisher's exact tests to compare categorical variables. *P*-value was considered significant if <0.05. Dataset available upon reasonable request contacting the corresponding author.

## Results

During the period analyzed, a total of 785 children younger than 5 years old diagnosed with malaria have been evaluated. Details are reported in Table 1.

**Table 1:** Malaria diagnoses in children younger than 5 years of age.

	**2018**		**2019**		**2020**		**2018 vs. 2020**	**2019 vs. 2020**
	**Males**	**Females**	**Males**	**Females**	**Males**	**Females**		
Jan	18	17	25	21	27	24	No COVID-19 In Sierra Leone	No COVID-19 In Sierra Leone
Feb	15	15	31	24	35	40	No COVID-19 In Sierra Leone	No COVID-19 In Sierra Leone
Mar	28	30	20	36	34	34	*p* > 0.05	*p* > 0.05
April	16	20	30	30	15	18	*p* > 0.05	*p* 0.01 (CI 13.5; 95% CI 7.05–19.95)
May	25	31	28	26	32	40	*p* > 0.05	*p* > 0.05

In March 2020, when the first cases of SARS-CoV-2 were reported in neighboring countries (Guinea and Liberia) but not in Sierra Leone, the number of cases was similar to those reported during the same month of the years 2018 and 2019.

A significant reduction of malaria diagnosis was noted on April 2020 (15 males, 18 females) compared with April 2019 (15 males, 15 females) (*p* 0.01; confidence interval 13–5; 95% confidence interval 7.05–19.95) but not compared with April 2018 (16 males, 20 females) (*P* > 0.05).

In May 2020, a new rise in malaria diagnoses was found (32 males, 40 females), compared with April 2020 (15 males, 18 females), but similar to May 2019 (28 males, 26 females) and 2018 (25 males, 31 females) (*P* > 0.05).

The number of malaria deaths in children younger than 5 years of age has been calculated from January 2018 to May 2020. The total numbers were similar: 9 deaths in 2018 (7 males, 2 females), 8 deaths in 2019 (3 males, 5 females), and 2 deaths (one male) detected from January to May 2020 (possibly a similar rate/year considering the short timeframe evaluated in 2020).

## Discussion

Our study allowed us to provide first data about the trends of malaria diagnoses in children younger than 5 years of age in three different years, showing that the number of diagnoses did not significantly change during COVID-19. Although an initial drop of malaria cases has been documented in April 2020, when Sierra Leone started the lockdown due to the first cases of COVID-19, we cannot exclude that it is a casual fluctuation in malaria diagnoses. Interestingly, when the lockdown was removed at the beginning of May 2020, a sudden increase in malaria diagnoses has been registered. Again, a causal relationship with the reduction of restrictive measures related to COVID-19 cannot be demonstrated but not even excluded.

According to local doctors and community health workers, in Sierra Leone, health authorities and health-care workers worked hard to ensure that people would not miss malaria care like what happened during the 2014–2016 Ebola virus disease (EVD) outbreak, when the number of malaria diagnoses, the number of patients with malaria seeking appropriate health care, and the volume of malaria treatments being dispensed dropped significantly compared with previous years (12, 13)^1^. It was estimated that ~7,000 additional malaria-associated deaths among children younger than 5 years in Guinea, Liberia, and Sierra Leone due to the Ebola outbreak [(12, 14) vecchie]. In the case of Ebola, the clinical similarity of EVD with malaria, fear of contracting Ebola in the health-care facilities, and interruption of distribution of insecticide-treated bed-nets were all contributing factors leading to disruption of malaria care (15).

Despite the beginning of April 2020, local health workers reported an initial reduction in malaria diagnosis, mainly due to fear of contracting COVID-19 in the health centers; after several health education activities, people came back to the health facilities when needed. In particular, during World Malaria Day on April 25th, an active campaign has been established by the government with the active involvement of community health workers, aiming to prevent interruption of malaria control programs due to COVID-19 (12). In particular, the campaign focused on:

- Education and information strategies aimed to eliminate the perception that health staff is infecting people with COVID-19
- Continuous health education on the use of insecticide-treated net, which has helped greatly in the reduction of positive malaria cases
- Implementation of malaria intermittent preventive treatment in infants given during the routine immunization activities to infants aged 10 weeks−9 months
- Nationwide free distribution of insecticide-treated bed-nets given to every household in May 2020, whereas this campaign has been interrupted during the previous Ebola outbreak
- Systematic malaria diagnostics as part of fever management and measures for early detection and treatment of malaria, including presumptive malaria treatment
- Implementation of community-based health workers for social engagement and monitoring of peripheries
- Continuation of the malaria drug and test supply.

In fact, on May 2020, when COVID-19 cases were still increasing in the country, the number of malaria diagnoses in our center in children younger than 5 years of age rose as well. To achieve this result, the Sierra Leone Government took preventive strategies after the lessons learned during the EVD outbreak.

This experience in a local health center previously involved by the EVD shows how a proactive approach may help to keep appropriate care for major killers in Africa, performing proactive screening to diagnose not only COVID-19 but also malaria. This is a necessary step in Sub-Saharan Africa because a missed diagnosis of malaria bears significant public health consequences for the community allowing the further spread of malaria, similarly to a missed diagnosis of COVID-19 (16). The clinical presentation of malaria is similar to COVID-19; moreover, both can be asymptomatic or paucisymptomatic or even present with a critical systemic disease (16). This further emphasizes the need to actively look for both diagnoses in malaria-endemic countries.

We are aware that our report has several limitations to address. The retrospective nature is a limitation itself. Moreover, we have been able to collect absolute numbers, and no comprehensive epidemiological/demographic data from the health center are currently available because the pandemic and the related restrictions are creating a higher workload for local workers and, at the same time, limits their possibility of interacting with other offices. Moreover, the restrictive measures lead to local organizational changes, including a reduction of the number of health workers concomitantly working at each health post, to reduce the risk of contagion of local workers, already extremely low in the area. Therefore, there were insufficient human resources and time to allow us a timely and more detailed data collection. Also, ecological changes during the three study periods may have potentially influenced the trend of local malaria diagnoses, although currently, there are no published pieces of evidence of unusually different rainy or dry seasons in the study period. Last, our data reflect the results of a national campaign in a single health center in Sierra Leone; therefore, these data cannot be generalized to the whole country or, in general, to Sub-Saharan Africa.

In conclusion, our preliminary data seem to suggest that a proactive approach in the management of malaria in endemic countries during COVID-19, including educational activities to sensitize the local population, is important and may be potentially useful to guarantee successful malaria diagnosis and treatment. However, long-term observational studies, including more centers, and particularly the peripheral health centers of LMICs, are needed to understand if a proactive and educational approach will be sufficient to prevent an excess of malaria deaths due to disruption of the local health systems related to the SARS-CoV-2 pandemic.

## Data Availability Statement

The raw data supporting the conclusions of this article will be made available by the authors, without undue reservation.

## Ethics Statement

The studies involving human participants were reviewed and approved by Bureh Town Community Hospital. Written informed consent to participate in this study was provided by the participants' legal guardian/next of kin.

## Author Contributions

DB, SS, and FI conceptualized the study. BC and FR collected data and performed the analyses. WR supervised the study team. All authors contributed to manuscript writing, read, and accepted the last version of the manuscript.

## Conflict of Interest

The authors declare that the research was conducted in the absence of any commercial or financial relationships that could be construed as a potential conflict of interest.

## Footnotes

^1^Available online at: http://blogs.biomedcentral.com/bugbitten/2020/04/17/malaria-during-the-covid-19-pandemic/ (accessed June 4, 2020).

## References

[1] Perisetti A Gajendran M Boregowda U Bansal P Goyal H. COVID-19 and gastrointestinal endoscopies: current insights and emergent strategies. Dig Endosc. (2020) 32:715–22. 10.1111/den.13693 32281689 PMC7262209

[2] Perisetti A Gajendran M Mann R Elhanafi S Goyal H. COVID-19 extrapulmonary illness - special gastrointestinal and hepatic considerations. Dis Mon. (2020) 66:101064. 10.1016/j.disamonth.2020.101064 32807535 PMC7386425

[3] Kopel J Perisetti A Roghani A Aziz M Gajendran M Goyal H Racial and gender-based differences in COVID-19. Front Public Health. (2020) 8:418 10.3389/fpubh.2020.00418 32850607 PMC7399042

[4] El-Sadr WM Justman J Africa in the path of Covid-19. N Engl J Med. (2020) 383:e11 10.1056/NEJMp2008193 32302075

[5] Buonsenso D Cinicola B Raffaelli F Sollena P Iodice F. Social consequences of COVID-19 in a low resource setting in Sierra Leone, West Africa. Int J Infect Dis. (2020) 97:23–6. 10.1016/j.ijid.2020.05.104. [Epub ahead of print]. 32497794 PMC7263219

[6] Adepoju P. Tuberculosis and HIV responses threatened by COVID-19. Lancet HIV. (2020) 7:e319–20. 10.1016/S2352-3018(20)30109-0 32277870 PMC7195157

[7] Buonsenso D Cinicola B Kallon MN Iodice F. Child healthcare and immunizations in sub-saharan africa during the COVID-19 pandemic. Front Pediatr. (2020) 8:517. 10.3389/fped.2020.00517 32850565 PMC7424001

[8] Buonsenso D Iodice F Sorba Biala J Goletti D. COVID-19 effects on tuberculosis care in Sierra Leone. Pulmonology. (2020) 10.1016/j.pulmoe.2020.05.013 32561353 PMC7275172

[9] Migliori GB Thong PM Akkerman O Alffenaar JW Álvarez-Navascués F Assao-Neino MM. Worldwide effects of coronavirus disease pandemic on tuberculosis services, January-April 2020. Emerg Infect Dis. (2020) 26:2709–12. 10.3201/eid2611.203163 32917293 PMC7588533

[10] Parri N Lenge M Cantoni B Arrighini A Romanengo M Urbino A. COVID-19 in 17 Italian pediatric emergency departments. Pediatrics. (2020) 146:e20201235. 10.1542/peds.2020-1235 32968031

[11] Weiss DJ Bertozzi-Villa A Rumisha SF Amratia P Arambepola R Battle KE. Indirect effects of the COVID-19 pandemic on malaria intervention coverage, morbidity, and mortality in Africa: a geospatial modelling analysis. Lancet Infect Dis. (2020) 10.1016/S1473-3099(20)30700-3. [Epub ahead of print]. 32971006 PMC7505634

[12] Wang J Xu C Wong YK He Y Adegnika AA Kremsner PG. Preparedness is essential for malaria-endemic regions during the COVID-19 pandemic. Lancet. (2020) 395:1094–6. 10.1016/S0140-6736(20)30561-4 32192582 PMC7158917

[13] Plucinski MM Guilavogui T Sidikiba S Diakité N Diakité S Dioubaté M. Effect of the Ebola-virus-disease epidemic on malaria case management in Guinea, 2014: a cross-sectional survey of health facilities. Lancet Infect Dis. (2014) 15:1017–23. 10.1016/S1473-3099(15)00061-4 26116183 PMC4669675

[14] Parpia AS Ndeffo-Mbah ML Wenzel NS Galvani AP. Effects of response to 2014–2015 Ebola outbreak on deaths from malaria, HIV/AIDS, and tuberculosis, west Africa. Emerg Infect Dis. (2016) 22:433–41. 10.3201/eid2203.150977 26886846 PMC4766886

[15] Walker PGT White MT Griffin JT Reynolds A Ferguson NM Ghani AC. Malaria morbidity and mortality in Ebola-affected countries caused by decreased health-care capacity, and the potential effect of mitigation strategies: a modelling analysis. Lancet Infect Dis. (2015) 15:825–32. 10.1016/S1473-3099(15)70124-6 25921597 PMC4824180

[16] Chanda-Kapata P Kapata N Zumla A. COVID-19 and malaria: a symptom screening challenge for malaria endemic countries. Int J Infect Dis. (2020) 94:151–3. 10.1016/j.ijid.2020.04.007 32344326 PMC7184246

